# Investigating The Role of Novel Bioactive Compound from *Ficus Virens* Ait on Cigarette Smoke Induced Oxidative Stress and Hyperlipidemia in Rats

**Published:** 2017

**Authors:** Danish Iqbal, Amir Khan, Irfan A Ansari, M. Salman Khan

**Affiliations:** a *Clinical Biochemistry and Natural Product Research Laboratory, Department of Biosciences, Integral University Lucknow-226026, India. *; b *Department of Medical Laboratory Sciences, College of Applied medical Sciences, Majmaah University, Al-majma’ah-11952, Saudi Arabia. *; c *Department of Maxillofacial Surgery (Biochemistry), College of Dentistry, Taif University, KSA.*

**Keywords:** Cigarette smoke, Oxidative stress, Hyperlipidemia, *Ficus virens*, Bioactive compound

## Abstract

The present study is premeditated to extenuate the role of *Ficus virens* extract and its bioactive compound on cigarette smoke, an important risk factor for CVD, induced oxidative stress and hyperlipidemia. Cigarette smoke (CS) exposure to rats results in significant loss of body weight and increases blood carbon monoxide saturation (carboxyhemoglobin), nicotine, plasma TC, TG, and LDL-C levels but reduced level of antiatherogenic HDL-C. Moreover, owing to substantial oxidative stress generated in rats due to cigarette smoke a significant increase in plasma and erythrocytes lipid peroxidation products were observed which was well correlated with increase in *ex-vivo *BDC (48%) and MDA (53%) level (p < 0.001). Simultaneous administration of FVBM extract at higher dose (100 mg/rat) and F18 (n-Octadecanyl-O-α-D-glucopyranosyl(6’→1’’)-O-α-D-glucopyranoside) compound to CS-exposed rats effectively blocked the increase in plasma lipid and lipoprotein levels (p < 0.001) which was due to the marked suppression in the hepatic HMG-CoA reductase activity (p < 0.001) and significantly inhibit the lipid peroxidation process thus preventing the membrane damage, LDL oxidation, and in turn subsequent atherosclerosis. Thus, the results clearly demonstrated the protective role of FVBM extract and F18 compound in risk factor induced cardiovascular disease.

## Introduction

Cardiovascular diseases (CVD) contribute around 17.3 million deaths globally (1). Cigarette smoking is one of the major risk factor for CVD and is the leading cause of preventable death and a major public health concern (1). Majority of compounds in cigarette smoke such as nicotine and carbon monoxide (CO) have been reported to further increase the risk of CVD in chronic smokers (2). Smoking can raise the cholesterol and free fatty acid concentrations in blood by increasing plasma total cholesterol (TC), triglycerides (TG), and low density lipoprotein-cholesterol (LDL-C) including Apo-B and decreasing the cholesterol and ApoA-1 level of high density lipoprotein (HDL) (3). Significant generation of free radicals and subsequent oxidative stress during smoking causes lipid peroxidation, LDL oxidation and decreased levels of antioxidants in the plasma of smokers (4) that ultimately results in CVD (3). Cigarette smokers encounter a sustained free radical load that can contribute to the oxidation of LDL *in-vitro *(5, 6), although data are conflicting (7). Moreover, erythrocytes from CS induced-hypercholesterolemia or oxidative stress may result in accelerated peroxidation reactions, cellular aberration, and alterations in lipid and protein structure (8). Thus, natural compounds with antioxidant properties contribute the protection of cell and tissue against deleterious effects caused by CS generated reactive oxygen species (ROS). Several natural antioxidants have been experimentally proved as protective agents against smoke induced-oxidative stress and hyperlipidemia (9-11). *Ficus* species due to their strong antioxidant and biological properties are known previously to diffuse the toxic free radical and can be used as a possible protective agent for treatment of oxidative stress related disorders (12-14). We previously described and documented that *Ficus virens* bark methanolic (FVBM) extract contained large amount of antioxidant with significant hypolipidemic property (15, 16), the work presented in this manuscript extenuate the protective role of FVBM extract and its principal bioactive compound, n-Octadecanyl-O-α-D-glucopyranosyl(6’→1’’)-O-α-D-glucopyranoside in CS-induced oxidative stress and hyperlipidemia.

## Experimental

*Chemicals*

Bradford dye (Sigma Aldrich, India), hydrogen peroxide (H_2_O_2_), isopropeol, glacial acetic acid (Merck Pvt Ltd, India), hemoglobin assay kit, total cholesterol (TC) and triglycerides (TG) kits were procured from Span Diagnostics Ltd. (India). Rodent Chow (Ashirwad pellets), capston cigarette (Capston, India) and all other chemicals were procured from Himedia Laboratories, Mumbai, India. All other chemicals and solvents used in this study were of analytical grade.

*Isolation of Bioactive compound*

Bioactive compound; n-Octadecanyl-O-α-D-glucopyranosyl(6′→1″)-O-α-D-glucopyranoside (F18) from FVBM extract has been isolated according to Iqbal *et al*. (16).

*Animals*

Male Sprague-Dawley (SD) rats weighed around 100-150 gm were procured from Indian Institute of Toxicology Research Center, Lucknow. The study protocol was approved by Institutional Animal Ethics Committee (IAEC) (registration number: IU/Biotech/project/CPCSEA/13/11). The rats have been housed 5 per cage for one week in the animal house for acclimatization at a temperature of 21-22 ˚C with 12 h light and dark cycle. The rats were given standard diet and water *ad libitum*.

*Dose preparation*

Sequentially extracted FVBM extract, its bioactive fraction (F18) and reference drug atorvastatin were dissolved in 10% dimethyl sulfoxide (DMSO) at different concentrations and were homogenized with saline. The doses of the extracts were selected on the basis of previously published reports (16-18).

*Diet/exposure to cigarette smoke*

FVBM extract, its bioactive fraction (F18) and atorvastatin suspension was administrated through gastric intubation in two divided doses (morning and evening) of 0.5 mL each/rat/day. Rats in smoking control group received 0.5 mL of saline containing 10% DMSO (vehicle) twice daily while rats in normal control group received 0.5 mL of saline containing 10% DMSO twice daily. The rats were divided randomly and equally (5 rats in each group) in groups as illustrated in Table 1. Rats were exposed to cigarette smoke in morning by keeping two rats in bottomless metallic container (10 ×11 × 16 inch), having two holes of 3 and 1.5 cm diameter, one on the either side. A burning cigarette was introduced through one hole (3 cm) and the other hole (1.5 cm) was used for ventilation. Animals were exposed to CS for 30 minutes, daily for 4 weeks with interval of 10 min between each 10 min exposure, using 3 cigarettes/day/2 rats in each group (11).

**Table 1:** Protocol for the treatment of cigarette smoke induce hyperlipidemia in rats.

**Group**	**Treatment**
N-C	Normal control
S-C	Cigarette smoking control + vehicle
FVT-1	Smoke-exposed + plant extract (FVBM) (50 mg/rat/day)
FVT-2	Smoke-exposed + plant extract (FVBM) (100 mg/rat/day)
CT	Smoke-exposed + bioactive compound (F18) (1 mg/rat/day)
AT	Smoke-exposed + standard (Atorvastatin) (1 mg/rat/day)

**Table 2:** Average body weight of rats in each group before and after 4 weeks of FVBM extract, F18 bioactive compound and atorvastatin treatment

**Group** *	**Before treatment**	**After treatment**
N-C	125.14 ± 2.67	130.85 ± 5.63
S-C	135.85 ± 3.93	80.56 ± 3.14 ^a^
FVT-1	125.28 ± 7.86	90.28 ± 3.14 c
FVT-2	133.46 ± 6.07	115.57 ± 4.45 ^a^
C-T	122.24 ± 7.86	125.28 ± 5.14 ^a^
A-T	126.78 ± 6.07	120.57 ± 5.45 ^a^

N-C: normal control, S-C: smoke-exposed control, FVT-1: fed 50 mg FVBM extract/rat/day, FVT-2: fed 100 mg FVBM extract/rat/day, C-T: fed 1 mg F18 bioactive compound/rat/day and A-T: given 1 mg atorvastatin/rat/day for 4 weeks.

* Values are mean (grams) SD from 5 rats in each group.

a Significantly different from N-C at p < 0.001.

a Significantly different from S-C at p < 0.001.

c Significantly different from S-C at p < 0.05.

**Table 3:** Impact of FVBM extract, F18 bioactive compound and atorvastatin on blood haemoglobin, carbon monoxide saturation and nicotine in cigarette smoke-exposed rats after 4 weeks of treatment

**Group** ^*^	**Haemoglobin** ** (g/dL)**	**Carbon monoxide** **saturation (SCO%)**	**Nicotine** **(µg/mL)**
N-C	14.86 0.764	3.26 0.089	0.998 0.012
S-C	11.60 0.515 (-21.9%)^b^	5.78 0.095 (+77.3%)^a^	2.16 0.033 (+116.4%) ^a^
FVT-1	12.35 0.617 (+6.4%)^c^	5.16 0.080 (-10.7%) ^a^	2.11 0.024 (-2.3%)^d^
FVT-2	14.62 0.721 (+26.1%) ^b^	4.07 0.091 (-29.6%) ^a^	1.48 0.042 (-31.5%) ^a^
C-T	14.74 0.732 (+27.1%) ^b^	4.76 0.078 (-17.6%) ^a^	1.59 0.035 (-26.4%) ^a^
A-T	13.99 0.677 (+20.6%) ^b^	3.97 0.043 (-31.3%) ^a^	1.98 0.022 (-8.3%)^b^

* Values are mean SD from blood of 5 rats in each group.

b Significantly different from N-C at p < 0.01.

a Significantly different from S-C at p < 0.001

b Significantly different from S-C at p < 0.01.

c Significantly different from S-C at p < 0.01.

d Non-significantly different from S-C at p > 0.05.

**Table 4:** Effect of FVBM Extract, F18 bioactive compound and atorvastatin on the ratios of plasma HDL-C/TC, HDL-C/LDL-C, TC/HDL-C and LDL-C/HDL-C in cigarette smoke-exposed rats after 4 weeks of treatment

**Group** ^*^ **/Ratio** ^†^	**HDL-C/TC**	**HDL-C/LDL-C**	**TC/HDL-C**	**LDL-C/ HDL-C**
N-C	0.35 ± .015	0.70 ± 0.031	2.85 ± 0.105	1.43 ± 0.057
S-C	0.08 ± 0.003 (-4.4 f)^a^	0.12 ± 0.006 (-5.8 f) ^a^	12.51 ± 0.46 (+4.4 f) ^a^	8.38 ± 0.35 (+5.9 f)^a^
FVT-1	0.18 ± 0.008 (+2.3 f) ^a^	0.30 ± 0.014 (+2.1 f) ^a^	5.55 ± 0.24 (-2.3 f) ^a^	3.33 ± 0.186 (-2.5 f) ^a^
FVT-2	0.30 ± 0.013 (+3.8 f) ^a^	0.58 ± 0.026 (+4.8 f) ^a^	3.33 ± 0.135 (-3.8 f) ^a^	1.73 ± 0.079 (-4.8 f) ^a^
C-T	0.33 ± 0.016 (+4.4 f) ^a^	0.65 ± 0.029 (+5.8 f) ^a^	3.04 ± 0.091 (-4.4 f) ^a^	1.53 ± 0.061 (-5.9 f) ^a^
A-T	0.33 ± 0.015 (+4.1 f) ^a^	0.64 ± 0.028 (+5.3 f) ^a^	3.03 ± 0.129 (-4.1 f) ^a^	1.56 ± 0.064 (-5.4 f) ^a^

N-C: normal control, S-C: smoke-exposed control, FVT-1: fed 50 mg FVBM extract/rat/day, FVT-2: fed 100 mg FVBM extract/rat/day, C-T: fed 1 mg F18 bioactive compound/rat/day and A-T: given 1 mg atorvastatin/rat/day for 4 weeks.

**Figure 1:**
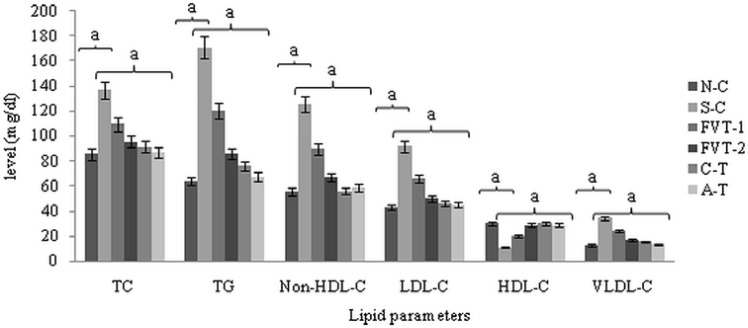
Effect of FVBM extract, F18 bioactive compound and atorvastatin on plasma triglycerides, total cholesterol, non-HDL-cholesterol, LDL-C, HDL-C and VLDL-C in cigarette smoke-exposed rats after 4 weeks of treatment.

^†^For the calculation of non-HDL-C, data is taken from Figure 1. ^*^Values are mean (mg/dL) SD from plasma, LDL-C, HDL-C and VLDL-C of 5 rats in each group. Significantly different from N-C at ^a^p < 0.001. Significantly different from S-C at ^a^p < 0.001.

† For the calculation of ratios, data is taken from Figure 1.

* Values are mean (ratio) SD from plasma of 5 rats in each group.

a Significantly different from N-C at p < 0.001.

a Significantly different from S-C at p < 0.001.

**Table 5:** Antioxidant impact of FVBM, F18 bioactive compound and atorvastatin on plasma total antioxidants, CD, LOOH and MDA contents in cigarette smoke-exposed rats after 4 weeks of treatment

**Group** ^*^	**CD**	**LOOH**	**MDA**	**Total antioxidants**
N-C	2.86 ± 0.141	1.02 ± 0.022	1.24 ± 0.015	85.47 ± 3.46
S-C	7.52 ± 0.325 (+162.9%)^a^	2.88 ± 0.104 (+182.4%) ^a^	2.68 ± 0.125 (+116.1%) ^a^	44.21 ± 2.04 (-48.27%) ^a^

FVT-1	5.28 ± 0.216 (-29.8%) ^a^	1.96 ± 0.083 (-31.9%) ^a^	1.95 ± 0.098 (-27.2%)^b^	56.42 ± 2.79 (+27.6%) ^a^

FVT-2	3.21 ± 0.110 (-57.3%) ^a^	1.29 ± 0.034 (-55.2%) ^a^	1.46 ± 0.062 (-45.5%) ^a^	81.53 ± 3.16 (+84.4%) ^a^

C-T	2.91 ± 0.108 (-61.3%) ^a^	1.08 ± 0.028 (-62.5%) ^a^	1.28 ± 0.042 (-52.2%) ^a^	79.18 ± 3.73 (+79.1%) ^a^

A-T	5.74 ± 0.209 (-23.7%)^b^	2.28 ± 0.026 (-20.8%) ^a^	2.14 ± 0.044 (-20.1%) ^c^	51.79 ± 2.92 (+17.1%) ^a^


N-C: normal control, S-C: smoke-exposed control, FVT-1: fed 50 mg FVBM extract/rat/day, FVT-2: fed 100 mg FVBM extract/rat/day, C-T: fed 1 mg F18 bioactive compound/rat/day and A-T: given 1 mg atorvastatin/rat/day for 4 weeks.

CD: Conjugated diene, LOOH: Lipid hydroperoxide, MDA: Malondialdehyde.

* Values are mean (µmole/dL) ± SD from plasma of 5 rats in each group.

a Significantly different from N-C at p < 0.001.

a Significantly different from S-C at p < 0.001.

b Significantly different from S-C at p < 0.01.

c Significantly different from S-C at p < 0.05.

**Table 6:** Basal MDA contents and its H_2_O_2_-induced MDA release in intact erythrocytes of cigarette smoke-exposed rats after 4 weeks of FVBM extract, F18 bioactive compound and atorvastatin treatment

**Group** *	**MDA** **content (nmole/g Hb)**	**MDA release(percent)**
N-C	5.67 ± 0.24	22.45 ± 0.92
S-C	12.74 ± 0.64 (+124.7%)^a^	46.6 ± 2.13 (+107.6%) ^a^

FVT-1	9.78 ± 0.46 (-23.2%) ^a^	34.26 ± 1.92 (-26.5%) ^a^

FVT-2	6.82 ± 0.31 (-46.4%) ^a^	29.87 ± 1.12 (-35.9%) ^a^

C-T	5.82 ± 0.23 (-54.3%) ^a^	23.68 ± 1.02 (-49.2%) ^a^

A-T	9.54 ± 0.32 (-25.1%) ^a^	36.81 ± 1.24 (-21.0%) ^a^


N-C: normal control, S-C: smoke-exposed control, FVT-1: fed 50 mg FVBM extract/rat/day, FVT-2: fed 100 mg FVBM extract/rat/day, C-T: fed 1 mg F18 bioactive compound/rat/day and A-T: given 1 mg atorvastatin/rat/day for 4 weeks.

CD: Conjugated diene, LOOH: Lipid hydroperoxide, MDA: Malondialdehyde.

* Values are mean (µmole/dL) ± SD from packed erythrocytes of 5 rats in each group.

a Significantly different from N-C at p < 0.001.

a Significantly different from S-C at p < 0.001.

b Significantly different from S-C at p < 0.01.

c Significantly different from S-C at p < 0.05.

**Table 7:** *Ex-vivo* and Cu^++^-catalyzed *in-vitro* oxidation of LDL, from plasma of cigarette smoke-exposed rats after 4 weeks of FVBM extract, F18 bioactive compound and atorvastatin treatment

**Group**	**LDL oxidation** ^+^				
	**Conjugated diene formation** *		**MDA content** ^#^		
	**Basal**	**Maximal**	**Lag Phase** **	**Basal**	**Maximal** ^¥^
N-C	178.49	945.86 (+430%)	88	5.43±0.212	15.26
S-C	264.36 (+48.3%)^†^	1348.56 (+42.6%)€	68 (-22.7%)¶	8.64±0.364^a^ (+53.2%)^†^	23.65 (+54.9%)€

FVT-1	234.72 (-11.4%)††	1264.35 (-6.2%)^α^	75 (+10.3%)§	7.43±0.202^ b^ (-14.0%)††	20.26 (-14.3%) ^#^

FVT-2	192.41 (-27.3%)††	1048.95 (-22.2%)^ α^	85 (+25.0%)§	6.24±0.244^ a^ (-27.8%)††	18.45 (-21.9%) ^#^

C-T	187.32 (-29.2%)††	989.35 (-26.6%)^ α^	87 (+27.9%)§	5.87±0.231^ a^ (-32.1%)††	16.6 (-29.8%) ^#^

A-T	244.54 (-7.5%)††	1294.28 (-4.0%)^ α^	73 (+7.4%)§	7.56±0.292^ c^ (-12.5%)††	20.43 (-13.6%) ^#^


N-C: normal control, S-C: smoke-exposed control, FVT-1: fed 50 mg FVBM extract/rat/day; FVT-2: fed 100 mg FVBM extract/rat/day, C-T, fed 1 mg F18 bioactive compound/rat/day and A-T: given 1 mg atorvastatin/rat/day for 4 weeks.

MDA: Malondialdehyde.

Significantly different from N-C at

* The CD values are expressed as nmole MDA equivalents/mg protein. Basal conjugated diene represent the *in-vivo* status of oxidized LDL.

** The lag phase is defined as the interval between the intercept of the tangent of the slope of the curve with the time expressed in minutes.

¥ The maximum *in-vitro* oxidation of LDL was achieved after 12 h of incubation with CuSo_4_ in each group,

† Percent increase with respect to basal value in N-C,

†† Percent decrease with respect to basal value in S-C,

¶ Percent decrease with respect to lag phase value in N-C,

§ Percent increase with respect to lag phase value in S-C,

€ Percent increase with respect to maximal value in N-C,

♯ Percent decrease with respect to maximal value in S-C, Significantly different from N-C at

a P < 0.001.

+ Values are obtained from LDL subpopulation, isolated from plasma of 5 rats in each group.

b p < 0.01, significantly different from S-C at

c p < 0.05.

**Figure 2:**
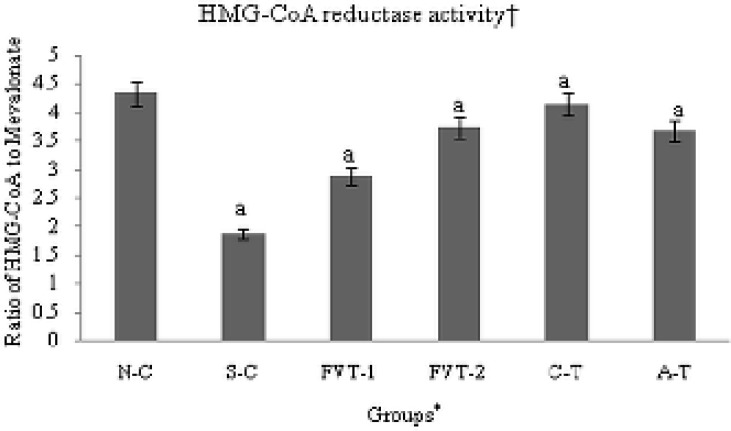
*In-vivo* regulation of hepatic HMG-CoA reductase activity in cigarette smoke-exposed rats treated with FVBM extract, F18 bioactive compound and atorvastatin for 4 weeks of treatment.

^†^Expressed as ratio of HMG-CoA to Mevalonate; lower the ratio higher the enzyme activity. ^*^Values are mean   SD from liver homogenate of 5 rats in each group. Significantly different from N-C at ^a^p < 0.001. Significantly different from S-C at ^a^p < 0.001.

*Collection of blood, plasma and packed erythrocytes*

At the end of the experiment, all the rats were anaesthetized and blood was collected in heparinized tubes by cardiac puncture. Plasma was collected from blood by centrifugation at 2,500 rpm for 30 min, aliquoted and stored at either 4 or −20 ˚C for future use.

Packed erythrocytes hemolysate was prepared as described by Lakshmi and Rajagopal (19). The packed erythrocytes obtained after the separation of plasma and buffy coat, were washed thrice with normal saline and a portion of washed erythrocytes was lysed in hypotonic (10 mM) sodium phosphate buffer, pH 7.4. A portion of the washed packed erythrocytes was stored at 4 ˚C for further use.

*Collection of organ and preparation of homogenate*

After the experiment, liver from the rats was promptly excised and chilled in ice-cold saline. After washing with saline, it was blotted and weighed. One g of wet tissues were cut into pieces and homogenized with 9 mL of chilled 0.1 M sodium phosphate buffer, pH 7.4 (containing 1.17% KCl) in a waring blender. The homogenate was centrifuged at 1,000 rpm for 10 min at 4 ˚C and finally was aliquoted and stored at −20 ˚C.

*Determination of hemoglobin (Hb) in blood*

Hemoglobin level was estimated by cyanmethemoglobin method of Drabkin and Austin [20] according to the procedure described in the instruction sheet enclosed with the reagent kit supplied by Span diagnostic. The percent blood hemoglobin was determined by measuring the absorbance of cyanomethemoglobin at 540 nm in eppendorf spectrophotometer using a hemoglobin standard.

*Determination of nicotine content in blood*

Nicotine content in blood samples was determined by the method of Varley *et al.* (21). Nicotine standard was treated in the similar manner and used in the calculation of nicotine present in the blood samples. 

*Determination of carbon monoxide saturation in a mixture containing hemoglobin and carboxy hemoglobin*

Carbon monoxide saturation (SCO) in blood samples was determined by the method of Varley *et al.* (21). The carbon monoxide saturation was calculated from the equation.

SCO% = (2.44 D^538^/ D^578^-2.68) 100

The constants 2.44 and 2.68 have been calculated from a series of 30 measurements of D^538^/ D^578^ to 0% COHb (100% Hb) and 100% COHb. The isobestic point was established at λ = 578 ± 0.5 nm (n = 100).

*Isolation of Plasma LDL and HDL*

The precipitation method described by Wieland and Seidel (22) was used for the isolation of plasma LDL and the method of Patsch *et al.* (23) was used for the isolation of HDL. 

*Measurement of ex-vivo and in-vitro Cu++-mediated susceptibility of LDL to oxidation*

The *ex-vivo* and *in-vitro* Cu^++^-mediated susceptibility of isolated LDL to oxidation was assessed by determining the lag phase of conjugated diene (CD) formation using the method of Esterbauer *et al.* (24, 25). Conjugated diene was calculated by using an extinction coefficient of 2.52×10^4^ M^-1^ cm^-1^ and expressed as nmole malondialdehyde (MDA) equivalent per mg LDL protein. The MDA content in LDL was assayed by the method of Niehaus *et al.* (26). The MDA concentration of the samples was calculated by using an extinction coefficient (1.56×10^5^ M^-1^cm^-1^).

*Measurement of plasma “total antioxidant power” (FRAP)*

The method of Benzie and Strain (27) was used for measuring the ferric reducing ability of plasma (FRAP assay) which estimates the “total antioxidant power”, with minor modification.

*Measurement of MDA release from intact erythrocytes*

The procedure of Cynamon *et al. *(28) was employed for the determination of MDA release from erythrocytes*. *

*Determination of MDA content in erythrocytes*

The determination of MDA in erythrocytes was carried out by adopting standardized protocol of Stocks and Dormandy (29). 

*Determination of plasma triglycerides and very low density lipoprotein-cholesterol*

Plasma TG was determined by using enzymatic kit (Merck, India) based on glycerol-3-phosphate oxidase peroxides (GPO-POD) method (30). The very low density lipoprotein-cholesterol (VLDL-C) in plasma was calculated by dividing plasma TG values (mg/dL) by a factor of 5 as described by Friedewald *et al*. (31).

*Determination of total cholesterol in plasma and lipoprotein*

Plasma TC, LDL-C and HDL-C were determined by using cholesterol enzymatic kit (Merck, India) based on cholesterol oxidase phenol aminophenazone (CHOD-PAP) method and the results were expressed as mg/dL. 

*Assay of HMG-CoA reductase activity in liver homogenate*

HMG-CoA reductase enzyme activity in liver homogenate was estimated indirectly by method of Rao and Ramakrishnan (32).

*Protein estimation*

The protein concentration of plasma, LDL and liver homogenate was analysed by the method of Bradford (33), using bovine serum albumin as standard. Aliquots were first precipitated with 10% TCA followed by centrifugation at 1500 rpm for 10 min. The pellets containing protein were dissolved in 0.5 N NaOH and suitable aliquots were used for protein determination.

*Data analysis*

For all assays, samples were analyzed in triplicate and the results were expressed as mean ± SD and the results were evaluated using one-way analysis of variance (ANOVA) and two tailed Students *t*-test. Statistical significance were expressed as *p < 0.05, **p < 0.01 and ***P < 0.001.

## Results

*Average body weight of rats in each group before and after 4 weeks of treatment*

As shown in Table 2, there was a significant decrease in body weight of smoke-exposed rats from 130.85 gm ± 5.56 in N-C to 80.56 gm ± 3.14 gm in S-C (p < 0.001) rats after 4 weeks of exposure to CS. Whereas, the average body weight of smoke-exposed rats treated with different doses of *F. virens *methanolic extract (FVBM-50 and FVBM-100), bioactive compound (F18), and atorvastatin was 90.28 ± 3.14, 115.57 ± 4.45, 125.28 ± 5.14, and 120.57 ± 5.45 (gm) (p < 0.001), respectively, which indicates a significant regain of average body weight when compared to S-C rats.

*Impact on hemoglobin (Hb), blood carbon monoxide saturation and blood nicotine in smoke-exposed rats treated for 4 weeks*

The results presented in Table 3, indicated the Hb, blood carbon monoxide saturation (carboxyhemoglobin) and blood nicotine level in N-C, S-C, and plant extract bioactive compound treated rats. Hemoglobin level was significantly reduced by 22% (p < 0.01) in smoke-exposed (S-C) rats, when compared to N-C value. However, treatment with higher dose of plant extract (100 mg/rat), purified compound (F18) and atorvastatin showed a highly significant increase in Hb concentration of 26%, 27% and 21% (p < 0.01) respectively, when compared to S-C rats. Both FVBM extract and F18 bioactive compound administration to smoke-exposed rats restored the Hb levels close to normal value. Furthermore, in S-C rats blood carbon monoxide saturation and blood nicotine levels were increased from 3.26 (SCO%) and 0.998 µg/mL in N-C to 5.78 (77%) and 2.16 µg/mL (116%) (p < 0.001) respectively. After 4 weeks of treatment blood carbon monoxide saturation and blood nicotine levels showed reduction of 11% (p < 0.001) and 2% (p < 0.05) in FVT-1; 30% (p < 0.001) and 32% (p < 0.001) in FVT-2, 18% (p < 0.001) and 26% (p < 0.001) in C-T and 31% (p < 0.001) and 8% (p < 0.01) in A-T rats, respectively, in comparison to values in S-C rats.

*Effect on plasma lipids and lipoprotein levels*

The results illustrated in Figure 1 showed that all the plasma lipids parameters, TC, TG, and non-HDL-C were significantly increased from 85.42, 64.05, and 55.53 mg/dL in N-C rats to 136.87, 171.05, and 125.93 mg/dL (p < 0.001) respectively in S-C rats. After 4 weeks of treatment with FVBM extract (50 and 100 mg/rat/day), levels of TC, TG and non-HDL-C were significantly decreased by 20%, 30%, 29% and 30%, 50%, 47% (p < 0.001) respectively, when compared to corresponding S-C values. Whereas, marked reduction of 33%, 55%, 56% and 36%, 61%, 54% (p < 0.001) was observed in TC, TG and non-HDL-C level of F18 and atorvastatin treated rats, when compared to corresponding values in S-C group.

Moreover, plasma LDL-C and VLDL-C levels were significantly increased from 42.7 and 12.8 mg/dL in N-C to 91.7 mg/dL (115%) and 34.2 mg/dL (167%) (p < 0.001) respectively, in S-C rats. After 4 weeks of treatment with FVBM extract (at higher dose), both LDL-C and VLDL-C levels showed a significant reduction of 46% and 50%, (p < 0.001) respectively, whereas, FVT-1 group exhibited much less reduction. Furthermore, LDL-C and VLDL-C level in C-T group were significantly reduced by 50% and 55% (p < 0.001) respectively, in comparison to corresponding values in S-C rats 

which was almost equivalent to the reduction observed in atorvastatin treated rats. Plasma HDL-C level were decreased from 30 mg/dL in N-C to 11 mg/dL (63%) (p < 0.001), in S-C values which was subsequently attenuated after the treatment with FVBM extract, bioactive compound and standard. Further, our result also depicted a significant decrease in HDL-C/LDL-C (5.8 fold) and HDL-C/TC (4.4 fold) ratio and a increase in TC/HDL-C (4.4 fold) and LDL-C/HDL-C (5.9 fold) ratios in CS-exposed hyperlipidemic rats (p < 0.001). The atorvastatin and bioactive compound treated rats exhibited marked increase of 4.1, 5.3 and 4.4, 5.8 fold (p < 0.001) in HDL-C/TC and HDL-C/LDL-C ratio (Table 4).

*Regulation of enzymatic activity of hepatic HMG-CoA reductase*

The result also exhibited a significant increase of 2.31 fold (p < 0.001) in hepatic HMG-CoA reductase activity; the rate limiting enzyme in the biosynthetic pathway of cholesterol when compared to N-C value (Figure 2). Among all the treated groups FVT-2 and C-T exhibited marked decline of 1.99 and 2.21 fold (p < 0.001) in HMG-CoA reductase activity, respectively (Figure 2), which was better or equivalent to the decline observed in atorvastatin treated rats.

*Impact on plasma total antioxidants and lipid peroxidation products*

Data in Table 5 demonstrate the antioxidant efficacy of FVBM extract, F18 bioactive compound and atorvastatin on *ex-vivo* plasma concentrations of total antioxidants, CD, LOOH, and MDA in CS-exposed rats. Data illustrated that cigarette smoke causes substantial decrease in plasma total antioxidants level and was reduced by 48% (p < 0.001) while CD, LOOH, and MDA levels were increased by 163%, 182%, and 116% (p < 0.001) respectively. Administration of FVBM extract (50 and 100 mg/rat/day), F18 and atorvastatin to smoke-exposed rats significantly increased the total antioxidants levels by 28%, 84%, 79% and 17% (p < 0.001), respectively, when compared to S-C value. On the other hand, CD, LOOH and MDA levels were significantly decreased by 30% (p < 0.001), 32% (p < 0.001) and 27% (p < 0.01) in FVT-1; 57%, 55% and 45% (p < 0.001) in FVT-2; 61%, 63% and 52% (p < 0.001) in C-T and 24% (p < 0.01), 21% (p < 0.001) and 20% (p < 0.05) in A-T rats, when compared to corresponding values in smoke-exposed rats.

*Effect on membrane lipid peroxidation in erythrocytes*

As seen in Table 6 , erythrocytes from smoke-exposed rats (S-C) group showed a greater susceptibility to hydrogen peroxide-­induced lipid peroxidation than those from N-C group. The MDA level was substantially increased by 125% (p < 0.001) in S-C rats, when compared to N-C value. Formation of MDA was markedly decreased by 23%, 46%, 54%, and 25% (p < 0.001) after the administration of FVBM extract, F18 and atorvastatin, respectively, when compared to the corresponding values in S-C. Similarly, H_2_O_2_-mediated release of MDA erythrocytes was increased from 22.45 in N-C to 46.6 nmol/gHb (108%) (p < 0.001) in S-C rats. A highly significant decrease of 27%, 36%, 50% and 21% (p < 0.001) in MDA release was seen in smoke-exposed rats treated with FVBM extract, F18, and atorvastatin, respectively, when compared to corresponding values in S-C rats.

*Antioxidant effect on basal and maximal level of CD formation and MDA content in LDL*

As depicted in Table 7, the *ex-vivo* basal CD level of LDL in smoke-exposed rats was increased by 48%, in comparison to the corresponding N-C values. Administration of FVBM-50, FVBM-100, F18 and atorvastatin to these smoke-exposed stressed rats partially blocked their *in-vivo *LDL oxidation and reduced their basal CD levels by 11%, 27%, 29% and 8%, respectively, in comparison to the corresponding S-C values. The maximal CD value of LDL in N-C was substantially increased by 430% in comparison to corresponding basal CD value in N-C. When compared to corresponding maximal CD value in N-C rats, LDL associated CD formation of S-C rats was increased by 43%.

Being a potent antioxidant, FVBM-100 and F18 significantly blocked the maximal CD concentration and reduced them by 22% and 27%, respectively, in comparison to corresponding maximal values in S-C rats. As expected, the lag phase time of LDL oxidation was reduced from 88 min in N-C to 68 min in S-C. Treatment of smoke-exposed rats with FVBM-50, FVBM-100, F18 and ATR, restored the lag phase time of LDL oxidation to 75 min, 85 min, 87 min, and 73 min, respectively. Similar to *ex-vivo* basal and *in-vitro* Cu^++^ catalyzed maximal CD values, the *ex-vivo* basal MDA content in LDL was significantly increased by 53% (p < 0.01) in S-C rats, when compared to corresponding values in N-C rats. FVBM-50, FVBM-100, F18 and atorvastatin treatment to smoke-exposed rats significantly blocked the *ex-vivo *increase in LDL MDA formation in S-C rats and reduced their levels by 14% (p < 0.01), 28% (p < 0.001), 32% (p < 0.001) and 13% (p < 0.05) respectively. An almost similar pattern was observed in maximal MDA content of LDL. 

## Discussion

Cigarette smoking (CS) is the foremost cause of morbidity and mortality worldwide (34, 35) and is considered to be the preventable risk factor for CVD (3). The current manuscript basically illustrates the use of FVBM extract and F18 compound in prevention and protection of CS-induced oxidative stress, hypercholesterolemia, and subsequent atherosclerosis. As shown in Table 2, there was significant decrease in body weight of smoke-exposed rats that may be due to reduced food intake or gastrointestinal irritation. Other reports also stated that smoker›s weight on average is about 4 kg less than non-smokers, mainly because of reduced food intake (36). Smoke-exposed rats treated with different doses of *Ficus virens *methanolic extract (FVBM-50 & FVBM-100), bioactive compound (F18) and atorvastatin indicates a significant regain of average body weight when compared to S-C rats.

The gas phase and tar of cigarettes contain free radicals, responsible for oxidative stress, has been hypothesized to be involved in the pathogenesis of smoking-related atherosclerosis (6). Moreover, cigarette smoking generates many toxic and carcinogenic compounds harmful to the health and was an unlikely cause for atherosclerosis, such as nicotine, nitrogen oxides, carbon monoxide (CO), hydrogen cyanide and free radicals (37-40 and 2). Similar to these reports, our results presented in Table 3, indicates blood carbon monoxide saturation and blood nicotine levels in smoke exposed rats were significantly increased and after 4 weeks a significant decrease was observed, in comparison to values in S-C rats. Moreover, both FVBM extract and F18 bioactive compound administration to smoke-exposed rats restored the Hb levels close to normal value. These results specify a strong protective effect of FVBM extract and F18 compound, which may help in lowering the menace of myocardial infarction in smokers.

Numerous smoking consequences have been described as being atherogenic, like direct vascular actions, oxidative stress, thrombogenic factors and secondary dyslipidemia (4, 40 and 41). In this context, our results illustrated that, all the plasma lipids parameters, TC, TG, non-HDL-C, LDL-C and VLDL-C levels were significantly increased (Figure 1). This increase in cholesterol and TG level of CS-exposed rats is in consensus with previously published reports (3, 42 and 43). Whereas, HDL-C level was significantly decreased by 63.4% (p < 0.001) in S-C rats when compared to N-C value (Figure 1). The increase in plasma TC level in S-C rats is apparently due to increased cholesterol synthesis (42-44), through the induction of hepatic HMG-CoA reductase activity-the rate limiting enzyme in the biosynthetic pathway of cholesterol. Chen and Loo (7) earlier reported that the increase in the cholesterol content of TG rich VLDL might be due to CS-induced reduction of lipoprotein lipase, which is responsible for the removal of TG from VLDL particles. Thus, the increased level of VLDL-C in S-C rats is also responsible for high concentrations of atherogenic cholesterol rich circulating LDL. The observed high level of VLDL-C in CS-exposed rats may responsible for reduced level of antiatherogenic HDL-C because of decreased availability of phospholipid remnants needed for the configuration of HDL from VLDL and a concomitant decline in lecithin cholesterol: acyltransferase (LCAT) activity.

Administration of FVBM extract at higher dose (100 mg/rat) and F18 bioactive compound to CS-exposed rats effectively blocked the increase in plasma lipid and lipoprotein level and reversed them to a level close to their normal control values, which is almost comparable to amelioration exhibited by standard drug atorvastation in cigarette smoke-exposed rats. Moreover, the present study observed diminished level of HDL-C in cigarette smoke-exposed rats when compared to normal rats which were in agreement with earlier reports (44, 45). Simultaneous administration of FVBM extract and F18 compound to CS-exposed rats significantly increases the HDL-C level which might be due to its profound antioxidant activity which in turn offers better protection to LDL as well as HDL from oxidative stress through its associated antioxidant enzyme paraoxonase (PON) (46). Consistent with earlier reports (47, 48) that established lipoprotein ratios, LDL-C to HDL-C and HDL-C to TC, are good forecaster for the existence and severity of CAD, our results depicted a significant decrease in HDL-C/LDL-C and HDL-C/TC ratio and a increase in TC/HDL-C and LDL-C/HDL-C ratios in CS-exposed hyperlipidemic rats. The atorvastatin and bioactive compound treated rats exhibited marked increase in HDL-C/TC and HDL-C/LDL-C ratio. An opposite pattern was observed in TC/HDL-C and LDL-C/HDL-C ratios. The results indicated a significant restoration of these ratios close to the normal values, which in turn implify the normalization of lipoprotein associated cholesterol level in CS-exposed rats treated with FVBM extract, F18 and atorvastatin. 

Enhanced plasma TC level in cigarette smoke-exposed rats is apparently due to significant amplification in hepatic HMG-CoA reductase activity-the rate limiting enzyme in the biosynthetic pathway of cholesterol (Figure 2). Moreover, the significant amelioration in plasma and lipoprotein lipid level exerted by these fractions was due to the marked suppression in the hepatic HMG-CoA reductase activity. Among all the treated groups FVT-2 and C-T exhibited marked decline in HMG-CoA reductase activity, respectively (Figure 2), which was better or equivalent to the decline observed in atorvastatin treated rats. These data suggest that FVBM extract/compound may exert their cholesterol lowering effect in CS-exposed hyperlipidemic rats via suppression of hepatic HMG-CoA reductase m-RNA expression which in turn cause inhibition of cholesterol synthesis (49). These data are in agreement with previous published reports that revealed hypolipidemic property of natural bioactive compound might be due to decrease in the efficiency of HMG-CoA reductase m-RNA translation and increasing the degradation of reductase protein post translationally (50-52). Our results represent an initial demonstration and exhibit strong rationale in support of the use of FVBM extract/bioactive compound, preferably F18, as a functional food in the prevention and treatment of tobacco induced dyslipidemia/hyperlipidemia and atherosclerosis.

It is well known that CS is associated with substantial increase in oxidative stress which is mainly due to increased lipid peroxidation or reduced antioxidants (3, 4). Throughout the course of CS-induced oxidative stress, oxygen derived free radicals like superoxide (O**¨**), hydrogen peroxide (H_2_O_2_), hydroxyl (•HO), peroxyl (ROO), alkoxy (RO) and nitric oxide (NO) are known to be generated in the cell, which can lead to oxidative modification of lipids and cause membrane damage, resulting in cell death (53, 4). It was previously found that serum MDA concentrations were higher in smokers and rats exposed to CS (54, 4). Similar to plasma, erythrocytes are also susceptible to damage by CS-induced oxidative stress because they are constantly exposed to both extracellular and intracellular sources of ROS and cause profound alteration in the structure and function of cell membrane that lead to cell death (8). Cigarette smoke is highly responsible for permanent inflammation and leads to imbalance in the profile of lipid peroxidation products (55), which can cause number of membrane changes including lipid peroxidation in CS-exposed erythrocytes of rats (8). The studies also showed that occupationally exposed human subjects indicate enhance lipid peroxidation and alter antioxidant systems in erythrocytes (56).

Consistent with these reports, our results demonstrated a significant increase in lipid peroxidation products in plasma and erythrocytes of subchronic cigarette smoke-exposed rats. The increase in plasma lipid peroxidation product is well correlated with a significant decline in plasma total antioxidant content which in turn is consistent with the prooxidant effect of CS in rats. These results are in consensus with previous finding (57), where an increase correlation between plasma MDA level and antioxidants has been reported in CS-exposed animals. 

However, protective role of lipid lowering agent with potent antioxidant property, such as FVBM extract and F18 compound, on the formation of lipid peroxidation products in plasma and erythrocytes of CS-exposed rats has not been reported. Our results showed that supplementation of FVBM extract and F18 bioactive compound to CS-exposed rats caused a significant decrease in plasma and erythrocytes lipid peroxidation products with a concomitant and significant increase in plasma total antioxidants and restored their levels close to corresponding control values in N-C (Table 6). These results are in concordant with our previously published *in-vitro* data that demonstrated the profound antioxidant property of FVBM extract and F18 bioactive compound (14, 16). Here it is interesting to mention that F18 compound, at a dose of 1 mg showed significant amelioration in reducing CS-induced oxidative stress parameters which were almost comparable to the FVBM extract at higher dose. The above data indicate that FVBM extract at higher dose and F18 compound strongly inhibit the lipid peroxidation process initiated by free radicals, thus preventing the membrane damage.

Several lines of research have established that lipoproteins play a pivotal role in atherogenesis (58, 59). Exposure of CS produces enhanced free radical that leads to the oxidative modification of lipoprotein (6). During oxidative modification native LDL is converted to Ox-LDL which is a key mediator of atherosclerosis and plasma concentrations has been found to be elevated in a range of cardiovascular diseases (60). 

Previous studies showed that elevated level of Ox-LDL concentrations in plasma have been found in smoke-exposed rats (61). The *ex-vivo *BDC levels and MDA have been suggested to reflect the *in-vivo *oxidation of LDL (62). The result demonstrated that LDL in the CS-exposed dyslipidemic rats had an increased susceptibility to oxidation when compared to LDL of normal rats. Moreover, our result showed that the *ex-vivo* BDC and MDA levels of LDL in CS-exposed hyperlipidemic rats were significantly increased. This increase susceptibility may result from increased oxidative stress, decrease total antioxidant and increase LDL cholesterol content. To the best of our knowledge no one to date has demonstrated the potent protective effect of *F. virens* extract or the isolated bioactive compound on the *ex-vivo* and *in-vitro* Cu^++^-catalyzed oxisability of plasma LDL of CS-exposed dyslipideamic rats. Simultaneous supplementation of FVBM extract and bioactive compound to CS-exposed rats blocked the *in-vivo* and *in-vitro* oxidation of LDL. As expected, the lag phase time of LDL oxidation was reduced and restored significantly after treatment. Similarly, *ex-vivo* basal and Cu^++ ^induced maximal formation of MDA in LDL was significantly decreased in CS-exposed rats treated with plant extract and the bioactive compound. The data summarises that FVBM extract at a lower dose was least effective as an antioxidant and was only able to partial restoration of the above lipid peroxidation parameters. The potent hypolipidemic property of FVBM extract (at higher dose) and the bioactive compound, as discussed above, is consistent with the excellent antioxidant property of these fractions.

The data summarises that FVBM extract at a lower dose was least effective as an antioxidant and was only able to partial restoration of the above lipid peroxidation parameters. The potent hypolipidemic property of FVBM extract (at higher dose) and the bioactive compound, as discussed above, is consistent with the excellent antioxidant property of these fractions.

## Conclusions

In conclusion our combined results clearly demonstrated the protective role of FVBM extract and F18 compound in risk factor induced cardiovascular disease. Moreover, the test fractions/pure compound exhibited significant protection against CS-induced severe oxidative stress and hyperlipidemia, which indicates that F18/FVBM extract are highly promising natural antioxidant and also can be used as an antiperoxidative, hypolipidemic, and antiatherogenic agent. However, further large scale clinical trials in hyperlipidemic smokers with and without coronary heart disease are required to substantiate their antioxidative, hypolipidemic, and atheroprotective properties.
